# Transposable Elements in the Organization and Diversification of the Genome of *Aegilops speltoides* Tausch (Poaceae, Triticeae)

**DOI:** 10.1155/2018/4373089

**Published:** 2018-09-26

**Authors:** Olga Raskina

**Affiliations:** Institute of Evolution University of Haifa, Aba-Hushi Avenue 199, Mount Carmel, Haifa 498838, Israel

## Abstract

Repetitive DNA—specifically, transposable elements (TEs)—is a prevailing genomic fraction in cereals that underlies extensive genome reshuffling and intraspecific diversification in the wild. Although large amounts of data have been accumulated, the effect of TEs on the genome architecture and functioning is not fully understood. Here, plant genome organization was addressed by means of cloning and sequencing TE fragments of different types, which compose the largest portion of the *Aegilops speltoides* genome. Individual genotypes were analyzed cytogenetically using the cloned TE fragments as the DNA probes for fluorescence in situ hybridization (FISH). The obtained TE sequences of the Ty1-*copia*, Ty3-*gypsy*, LINE, and CACTA superfamilies showed the relatedness of the *Ae. speltoides* genome to the Triticeae tribe and similarities to evolutionarily distant species. A significant number of clones consisted of intercalated fragments of TEs of various types, in which Fatima (Ty3-*gypsy*) sequences predominated. At the chromosomal level, different TE clones demonstrated sequence-specific patterning, emphasizing the effect of the TE fraction on the *Ae*. *speltoides* genome architecture and intraspecific diversification. Altogether, the obtained data highlight the current species-specific organization and patterning of the mobile element fraction and point to ancient evolutionary events in the genome of *Ae. speltoides*.

## 1. Introduction

Repetitive DNA—specifically, transposable elements (TEs)—constitutes at least 45% of the human genome, wherein the fraction of long interspersed nucleotide element (LINE) retrotransposons is 17% [1]. In plants, TEs comprise up to 80% of the genomes, with prevailing long terminal repeat (LTR) families of Ty1-*copia* and Ty3-*gypsy* retrotransposons [2, 3], which vary extensively in their sequence motifs and abundances, even between closely related species [4, 5]. Mobile elements move to new sites in the genome either through an RNA intermediate via a copy-and-paste mechanism (retrotransposons of Class I) or directly through a cut-and-paste mechanism (transposons of Class II) [1, 2, 6], generating the basis for genetic variability in somatic and generative tissues and resulting in intraspecific variations [7, 8]. TEs modify the host genome via insertional mutagenesis, affect both the expression of neighboring genes and translation, and contribute to new gene generation [9–12]. TE mobilization, especially under conditions of environmental stress and/or hybridization, causes prompt karyotype changes that accompany speciation [13–15].

Many epigenetically silent copies and fragments of TEs accumulate in the genome as an integral part of heterochromatin [10, 16], and the methylation and epigenetic remodeling of heterochromatin-specific repeats have been involved in the siRNA-mediated transcriptional silencing of full-length, transpositionally competent TEs [17, 18]. At the cytological level, heterochromatic DNA is traced as condensed chromatin blocks throughout the cell cycle, except during replication in the late S-phase [19]; the replication of euchromatic gene-rich DNA occurs earlier in the S-phase. Nuclear chromatin organization and dynamics are associated with genome functioning; during cell differentiation, gene replication and expression timing can change due to repositioning in the nuclei and chromatin remodeling [20]. Regardless of whether high polymorphism is present, the heterochromatin pattern is an integral chromosome- and species-specific characteristic. In the wild, ongoing chromosomal rearrangements lead to considerable changes in the numbers, sizes, and positions of highly repetitive DNA clusters and underlie the divergence of natural populations [21].

Despite the large amount of accumulated data, the significance of the complex repetitive DNA fraction in the eukaryotic genome restructuring and functioning is still not completely understood. Here, plant genome organization is addressed in terms of the genomic content and chromosomal patterns of different TE types. The present study was conducted using wild diploid (2*n* = 2*x* = 14) predominantly cross-pollinated, but self-compatible, goatgrass, *Aegilops speltoides* Tausch (sect. Sitopsis, Triticeae), which is considered the G- and B-genome ancestor of wild and cultivated allopolyploid wheats [22]. In natural panmictic populations, *Ae. speltoides* is presented with two morphs, ssp. *ligustica* (dominant) and ssp. *aucheri* (recessive) [23, 24]. In addition, plants with intermediate *ligustica*/*aucheri* phenotypes have been revealed in natural populations, suggesting genetic changes in the linked group of genes encoding the spike morphology [25]. *Ae. speltoides* has a large genome of 5.5–5.8 pg/1C [26], comprising an extraordinary number of TEs, especially LTR retrotransposons. In the wild, *Ae. speltoides* possesses a wide spectrum of chromosomal rearrangements and extranumerary B chromosomes [25, 27]. The next-generation 454 sequencing of the individual genotype showed that the predominant Ty1-*copia* superfamily exceeds 12% of the identified TEs in the *Ae. speltoides* genome and the Ty3-*gypsy* family Fatima makes up to 7% of the TEs [4]. Mobile elements are considered perpetual rebuilders of the *Ae. speltoides* genome, especially in stressful environments, and they are likely recruited into evolutionarily significant events, leading to population divergence and speciation at the diploid level and via allopolyploidy [28–30]. In the present study, the composition of the *Ae. speltoides* genome was investigated by means of cloning and sequencing of fragments of different TE types, which compose the largest portion of the genome and form the major fraction of heterochromatin. The data obtained from TE fragments' cloning and sequencing revealed different types of TEs, which showed the relatedness of *Ae. speltoides* to the Triticeae tribe and similarities to evolutionarily distant species. At the chromosomal level, different TE clones demonstrated sequence-specific patterning, highlighting the effect of the TE fraction on the *Ae*. *speltoides* genome architecture and intraspecific diversification.

## 2. Materials and Methods

### 2.1. Plant Material

Original plants of *Ae. speltoides* from contrasting allopatric populations from Cankiri (Turkey; PI 573448, USDA), Ankara (Turkey; PI 573452, USDA), and Katzir (Israel; 2.93, Institute of Evolution University of Haifa), line TS43 from Giv'at Koach (Israel; TS43, Weizmann Institute of Science), and artificial F_1_–F_2_ intraspecific hybrids of these plants [25] were analyzed using fluorescence in situ hybridization (FISH). The genotype F_1__K5/A2 carries one extranumerical B chromosome (2*n* = 2*x* = 14 + *B*) inherited from the K5 maternal genome. This set of plants was used to evaluate and characterize the TE chromosomal patterns under the native and artificial heterozygosity of the *Ae. speltoides*.

### 2.2. DNA Isolation and Polymerase Chain Reaction (PCR) Amplification of Reverse Transcriptase (RT) Gene Sequences of Ty1-*copia*, Ty3-*gypsy*, and LINE Retroelements

Genomic DNA was isolated from the young leaves of the individual TS43 genotype using the CTAB method [31]. Degenerate oligonucleotide primers were used for PCR amplification from the genomic DNA of conserved regions of the RT genes of the Ty1-*copia* [32], Ty3-*gypsy* [33], and LINE [34] retroelements. PCR amplifications were conducted in 25 *μ*l reaction volumes containing 12.5 *μ*l of DreamTaq™ Green PCR Master Mix (2x; Fermentas), 150–200 ng of genomic DNA from TS43 leaves, and each degenerate primer in a final concentration of 2 *μ*M. For PCR amplification of the individual TE clones, 0.5–1.0 ng of plasmid DNA was used as the template and standard T7 and SP6 primers for the pGEM®-T Vector (Promega, USA) were employed in a final concentration of 0.5 *μ*M. The PCR conditions were as follows: an initial denaturation for 4 min at 94°C, 35 cycles of amplification (30 s at 94°C, 1 min at 50°C, 1 min at 72°C), and a final elongation of 10 min at 72°C. The PCR-amplified fragments were purified using the HiYield Gel/PCR DNA Fragments Extraction Kit (RBC Bioscience, Taiwan).

### 2.3. The Cloning and Sequencing of PCR-Amplified TE Fragments

Total purified PCR-amplified products were ligated into a pGEM®-T Easy Vector and transformed into *Escherichia coli* JM109 blue competent cells according to the standard manufacturer's protocol (pGEM-T Easy Vector System II; Promega, USA). Recombinant clones were isolated using the PureYield™ Plasmid Miniprep System (Promega, USA), screened for inserts by PCR, and, following digestion with *Eco*RI (Supplementary Figure S1), sequenced with universal T7 primer. The obtained sequences of TE fragments, 34 in total, were deposited in the National Center for Biotechnology Information (NCBI) GenBank database with the accession numbers KY404239 (Aesp1) to KY404272 (Aesp34) (Supplementary Table S1).

The selected clones were PCR amplified using plasmid DNA as the template. They were used as the DNA probes for FISH.

### 2.4. Identification and Comparison of the Sequences

The sequences obtained in this study were analyzed for similarity to known sequences using the BLAST packages provided by the NCBI (https://blast.ncbi.nlm.nih.gov/Blast.cgi) [35], TRansposable Elements Platform (TREP; http://botserv2.uzh.ch/kelldata/trep-db/index.html) [36], SENSOR software (Genetic Information Research Institute (GIRI); http://www.girinst.org/) [37], and Rice Genome Annotation Project (http://rice.plantbiology.msu.edu/index.shtml) [38]. They were compared to each other using the NCBI ALIGN program and CLUSTALW software (Multiple Sequence Alignment by CLUSTALW; Kyoto University Bioinformatics Center; http://www.genome.jp/tools/clustalw).

### 2.5. Cytogenetic Analysis of the TEs' Chromosomal Patterns

For the FISH experiments, cytological slides of individual anthers and seedling shoot apical meristems containing well-spread chromosomal plates were used. The chromosome spreads, DNA probe labeling, and FISH procedures were conducted as previously described [39]. Tandem repeats Spelt1 [40], pTa71 (for the localization of 45S rDNA) [41], and As5SDNAE (for the localization of 5S rDNA) [42] were used as the DNA probes for FISH. The PCR-amplified fragments were purified using the HiYield Gel/PCR DNA Fragments Extraction Kit (RBC Bioscience, Taiwan) and used as the DNA probes in the standard oligolabeling protocol as previously described [39, 43]. The DNA probes were directly labeled with Cy-3, fluorescein-12-dUTP, and ATTO-425 (Jena Bioscience, Germany). AT-specific 4′,6-diamidino-2-phenylindole (DAPI) and GC-specific chromomycin A_3_ (CMA_3_) fluorochromes were used for differential staining to reveal AT-enriched heterochromatin patterns and GC-enriched heterochromatic clusters in the nuclear organizer regions (NORs) on chromosomes 1 and 6 in the *Ae. speltoides* genome. The slides were examined on a Leica DMR microscope equipped with a DFC300 FX CCD camera.

## 3. Results

### 3.1. Identification and Comparative Characterization of the Sequenced TE Fragments

Three sets of nucleotide sequences were obtained via the cloning and sequencing of PCR products amplified using degenerate primers for RT of Ty1-*copia* (10 sequences, clones Aesp1 to Aesp10), Ty3-*gypsy* (13 sequences, clones Aesp11 to Aesp23), and LINE (11 sequences, clones Aesp24 to Aesp34) retrotransposons (Supplementary Table S1). PCR with degenerate primers for RT of Ty1-*copia* amplified a mix of approximately 280 bp fragments (Supplementary Figure S1). Comparing the sequences with each other showed that the homology between clones Aesp1 to Aesp6 was 95-96% at 100% coverage (Supplementary Table S1). These sequences show high similarity (83–98%, coverage of 85–99%) to RT of the WIS family of Ty1-*copia* retrotransposons, and clone Asp7 showed the highest similarity to Angela. Clone Aesp8 shows 93% homology at 95% coverage to the Ty1-*copia* element Rada reported in the storage protein activator (spa) locus in *Ae. speltoides* and the *Triticum aestivum* and *T. durum* genomes. Clone Aesp9 was identified as exhibiting similarity to the Ty1-*copia* retroelement LeojygB_RLC_Hvul_LeojygB_Hn582D21 in *Hordeum vulgare*. The Aesp10 sequence showed 88% homology to the chromosome 3B of *T. aestivum* (accession no. HG6703064) and 64–70% similarity to the RT genes of *Oryza sativa*, *Zea mays*, and *Setaria italica* (Supplementary Table S1). Thus, six extremely similar sequences of the Ty1*-copia* type—Aesp1 to Aesp6—showed high identity to the WIS retrotransposon; Aesp7 was extremely similar to Angela; and three sequences—Aesp8, Aesp9, and Aesp10—significantly differed from each other and the group of Aesp1–Aesp7, demonstrating homology of 64–85% to non-Triticeae species, such as rice, foxtail millet, false brome, and bamboo.

**Figure 1:**
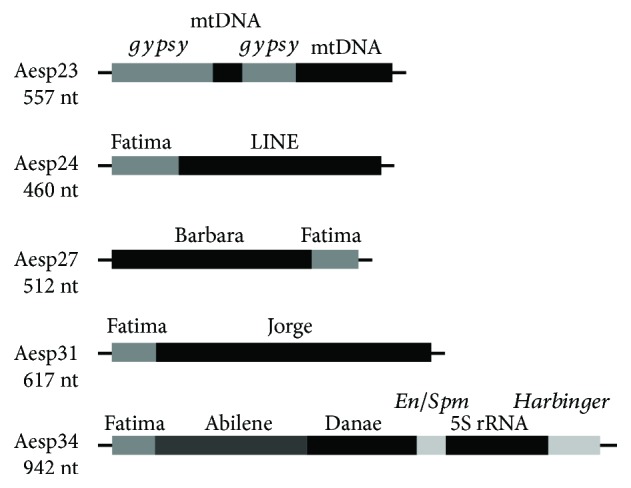
Structural organization of individual cloned transposable element (TE) sequences.

The cloning of the PCR-amplified fragments of Ty3-*gypsy* retrotransposons and following sequencing of randomly chosen colonies yielded a wide range of sequences of different lengths (Supplementary Figure S1). The analysis for similarity to known sequences showed that clones Aesp11 to Aesp16 (from 361 to 1006 nt in length) exhibited high similarity to the Fatima family of Ty3-*gypsy* retrotransposons; however, they differed from each other, except the Aesp11 and Aesp13 clones, which showed identity of 92%. In addition, the sequences Aesp11 and Aesp13 showed 93–97% similarity to the clones Gas-1 to Gas-5 of *Ae. speltoides*. Two clones, Aesp17 (505 nt) and Aesp18 (502 nt) showed significant homology to Ty3-*gypsy* retrotransposon Carmilla. Two almost identical clones, Aesp20 (997 nt) and Aesp21 (1001 nt), exhibited similarity to Ty3-*gypsy* of *Brachypodium distachyon* and 66–68% of homology at 88–89% coverage to the Ty3-*gypsy* VRN-B1 retrotransposon of allopolyploid wheat. One sequence, Aesp22 (202 nt), was identified as DNA transposon Jorge (TIR, CACTA) of Class II. Clone Aesp23 comprised two fragments showing 73–78% similarity to Ty3-*gypsy* of the chickpea (*Cicer arietinum*) and two fragments of mitochondrial DNA with high similarity to the mitochondrial genomes of *Ae. speltoides* and allopolyploid wheats (Figure 1; Supplementary Table S1). Therefore, the set of 13 clones comprised 11 sequences of Ty3-*gypsy*, wherein the Fatima family is presented by six sequences, two sequences were similar to Carmilla, one clone was similar to Nusif, and two sequences were similar to the VRN-B1 Ty3-*gypsy* retrotransposon. The Aesp23 sequence comprises fragments of Ty3-*gypsy* retroelements and mitochondrial DNA. One sequence was identified as CACTA transposon Jorge of Class II.

PCR with degenerate primers for RT of LINE elements and the following cloning and sequencing of randomly selected colonies yielded three clones, namely Aesp24 (460 nt), Aesp25 (384 nt), and Aesp26 (710 nt), comprising similar sequences to non-LTR LINE-like elements and short fragments similar to Ty3-*gypsy* Fatima (Supplementary Table S1, Figure S1). Four extremely similar clones, Aesp27 to Aesp30 (509–512 nt) comprised 404 nt of Ty1-*copia* element Barbara and a fragment of 82 nt of Ty3-*gypsy* Fatima (Figure 1). Two almost identical sequences, Aesp31 (617 nt) and Aesp32 (605 nt), comprised fragments comparable to DNA transposon Jorge (TIR, CACTA; 90–93% identity) and 81 nt of Ty3-*gypsy* Fatima. Clone Aesp33 (491 nt) showed 87–89% similarity to the Ty3-*gypsy* Fatima family. The Aesp34 (942 nt) sequence comprised three Ty3-*gypsy* fragments similar to Fatima (84 nt), Abilene (308 nt), and Danae (190 nt), a short fragment of 47 nt of the *En*/*Spm* (TIR, CACTA) transposon, a fragment of 201 nt of 5S rDNA, and a fragment of 94 nt of the *Harbinger* (TIR, *Harbinger*) transposon. In turn, the part of the Aesp34 sequence (540 nt) containing the fragments of Danae, *En*/*Spm*, 5S rDNA, and *Harbinger* exhibited 84% homology (at 100% coverage) to the genomic scaffold of chromosome 3B of *T. aestivum* (accession no. HG670306.1) and 70% homology to the cytokinin oxidase/dehydrogenase (CKX2.5) gene (accession no. JN381556). Thus, in this set of 11 clones, only three sequences were similar to non-LTR LINE elements, while four clones comprised fragments of Ty1-*copia* Barbara and Ty3-*gypsy* Fatima, one sequence was highly similar to Fatima, and two clones comprised fragments of CACTA transposons Jorge and Fatima. One clone, Aesp34, contained fragments of three different Ty3-*gypsy* elements, two fragments of DNA transposons, and a fragment of 5S (Figure 1; Supplementary Table S1).

### 3.2. Chromosomal Patterning of Individual TE Clones in the Genome of *Ae. speltoides*

**Figure 2:**
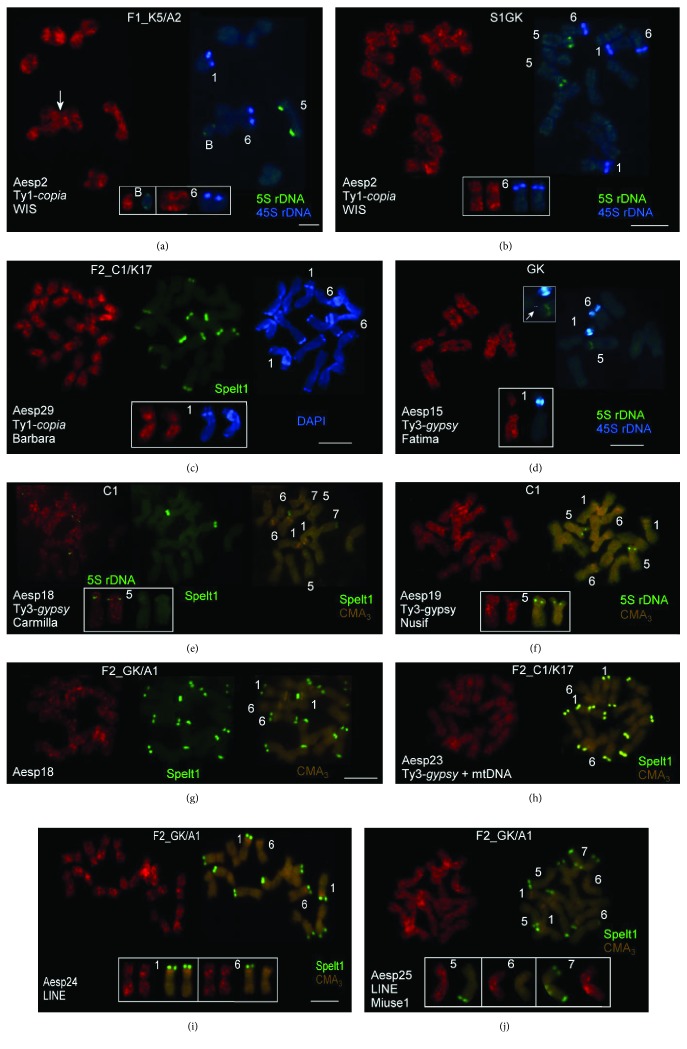
Fluorescent in situ hybridization (FISH) with individual cloned transposable element (TE) sequences on meiotic and somatic chromosomes of *Aegilops speltoides.* (a–b) Chromosomal distribution of the clone Aesp2 (WIS, Ty1-*copia*) on meiotic and somatic chromosomes of two different genotypes. (a) Meiotic chromosomes of the hybrid genotype F_1__K5/A2 obtained in the cross ♀Katzir-5 × ♂Ankara-2 (K5/A2). In the small box, supernumerary B chromosome and chromosome 6 forming bivalents are enlarged. In the B chromosome, large intercalary TE and small TE clusters adjacent to the 5S rDNA blocks are revealed in both arms. Large TE clusters in both arms in both instances of homologous chromosome 6 are detected. Nonhomologous synapses between chromosome 6 and another bivalent is indicated by an arrow. (b) Somatic chromosomes of the genotype S1GK achieved by self-pollination of the TS43 (Giv'at Koach (GK)) plant. In the small box, instances of homologous chromosome 6 are enlarged; TE clusters are detected in the long arms. (c) Chromosomal distribution of clone Aesp29 (Barbara, Ty1-*copia*) in the hybrid genotype F_2__C1/K17 obtained by self-pollination of the F_1__C1/K17 genotype; cross-combination ♀Cankiri-1 × ♂Katzir-17 (C1/K17). Homologous chromosomes 1 are enlarged in the small box. Significant depletion in TE abundance is observed in the pericentromeric and distal/terminal chromosomal regions, including DAPI-positive AT-enriched heterochromatic and Spelt1 clusters. (d) Distribution of the clone Aesp15 (Fatima, Ty3-*gypsy*) on meiotic chromosomes in the genotype GK (line TS43). Panchromosomal TE clustering is observed; homologous chromosomes are similar in their TE patterns. In the small box, the bivalent of chromosome 1 is enlarged. The nonhomologous synapsis between chromosomes 1 and 5 is shown with an arrow in the small upper box; the ectopic chromatin fiber between bivalents is shown with a dashed line. (e) Chromosomal pattern of clone Aesp18 (Carmilla, Ty3-*gypsy*) in the somatic chromosomes of genotype C1 (Cankiri-1). Intensive clustering throughout the whole chromosome lengths is observed. In the small box, instances of homologous chromosome 5 are enlarged. (f) Patterning of clone Aesp19 (Nusif, Ty3-*gypsy*) in the somatic chromosomes of genotype C1. The homologous chromosomes show similarity in TE patterning. In the small box, instances of chromosome 5 are enlarged. Differences in the chromosome 5′s morphology give evidence for heterozygosity in terms of rearrangement in the C1 genotype. (g) Distribution of clone Aesp18 (Carmilla, Ty3-*gypsy*) in genotype F_2__GK/A1 obtained by self-pollination of the F1_GK/A1 (cross-combination ♀GK × ♂Ankara-1 (GK/A1)). The TE pattern is comparable to the pattern observed in genotype C1 (e). (h) Dispersal distribution of clone Aesp23 (Ty3-*gypsy* + mitochondrial DNA) throughout the somatic chromosomes in genotype F_2__C1/K17. (i) Chromosomal patterning of clone Aesp24 (long interspersed nucleotide element (LINE)) in the F_2__GK/A1 genotype. Large clusters are observed in the intercalary regions. Chromosomes 1 and 6 are enlarged in the small box. (j) Distribution of clone Aesp25 (LINE) in the somatic chromosomes of the F_2__GK/A1 genotype. Rear large TE clusters are revealed in some chromosomes. In the small box, individual chromosomes 5, 6, and 7 are enlarged. Scale bar = 10 *μ*m.

The genomes of parental plants from Katzir (K5 and K17) and Giv'at Koach (GK) were highly enriched with Spelt1 tandem repeat clusters, in contrast to plants from Ankara (A1 and A2) and Cankiri (C1), and the F_1_ and F_2_ genomes were heterozygous for chromosomal markers, specifically, Spelt1 clusters [25]. The species-specific Spelt1 tandem repeat typically forms clusters in the distal/terminal heterochromatic chromosomal regions in *Ae. speltoides* (Figures 2(c), 2(e), 2(g)–2(j)).

The cytogenetic analysis displayed certain features in the chromosomal patterning of the different TE sequences obtained in this study. The FISH experiments revealed a panchromosomal distribution of Ty1*-copia*, Ty3-*gypsy*, and LINE retrotransposons in the *Ae. speltoides* genome and TE-sequence-specific clustering in certain chromosomal positions.

The Ty1-*copia* retroelement WIS (clone Aesp2) demonstrated dispersed distribution along the chromosomes' length, forming more prominent rare clusters in distal positions on meiotic (Figure 2(a)) and somatic (Figure 2(b)) chromosomes. Extranumerical B chromosome carried a large intercalary WIS cluster and small TE clusters adjacent to 5S rDNA clusters in both arms (Figure 2(a)). The Ty1-*copia* Barbara (clone Aesp29) showed predominant intercalary clustering and significant depletion in the distal/terminal and pericentromeric chromosome regions (Figure 2(c)), in contrast to WIS. The homologs demonstrated significant similarity in the Aesp2 and Aesp29 chromosomal patterns, as shown for individual chromosomes in the small boxes (Figures 2(a)–2(c)).

The Ty3-*gypsy* retrotransposons Fatima (Aesp15), Carmilla (Aesp18), and Nusif (Aesp19; Figures 2(d)–2(g)) demonstrated TE-sequence-specific chromosome patterning and formed numerous intercalary, distal/terminal, and pericentromeric clusters of different sizes and fluorescence intensities. In particular, small terminal clusters of Spelt1 and Carmilla were observed in the long arm of chromosome 5 (Figure 2(e)); differences in homologous chromosome 5 morphology indicated heterozygosity for rearrangements in the C1 genome. These three Ty3-*gypsy* clones flanked the regions of 5S rDNA and 45S rDNA, rather than intercalating in them.

Clone Aesp23, combining fragments of *gypsy* and mitochondrial DNA, demonstrated dispersed distribution throughout all the chromosomes (Figure 2(h)). Intensive clustering was detected in the intercalary, pericentromeric, and distal/terminal chromosome positions.

Two sequences of non-LTR LINE retrotransposons, clones Aesp24 and Aesp25, were dissimilar in their chromosomal distributions. Total fluorescence of Aesp25 appeared significantly lower, and the number of clusters was considerably less compared with Aesp24 (Figures 2(i)-2(j)); however, these rare clusters were extremely distinct.

Thus, all the TE clones demonstrated sequence-specific peculiarities in their distribution, with preferential clustering in certain chromosomal positions, specifically, in the distal/terminal and pericentromeric regions corresponding to DAPI- and Giemsa-positive [44] heterochromatic blocks in the *Ae. speltoides* genome. In many cases, homologous meiotic and somatic chromosomes demonstrated similarities in TE patterning. The same clone(s) showed similar chromosomal distribution/patterning in different genotypes, and in the same genotype(s), different TE clones demonstrated TE-sequence-specific distribution.

## 4. Discussion

In the present study, TE fragments of different classes and families were sequenced and cytologically visualized in the *Ae. speltoides* genome. The composition and pattern of repetitive DNA largely determine distinctiveness and reflect the evolution of the species. Various types of repetitive DNA constitute the genome of *Ae. speltoides*, wherein the fraction of mobile elements is the largest. Among the sequences of Ty1-*copia* obtained in this research (Supplementary Table S1), there were six fragments of WIS (clones Aesp1 to Aesp6) and one fragment of Angela (Aesp7) belonging to the BARE1 clade, which represents the largest TE portion of the *Ae. speltoides* genome [4]. The pairwise alignment of these clones and sequences in the publicly available databases suggests enrichments of the *Ae. speltoides* genome with highly diverged elements of the *copia* superfamily. In addition, four extremely similar sequences, Aesp27 to Aesp30, were classified as belonging to the Barbara family of LTR Ty1-*copia* retrotransposons. Clone Aesp9 was identified as similar to Ty1-*copia* retroelement LeojygB in *H. vulgare*, and clone Aesp8 was extremely different from all the other sequences, showing high homology to the Ty1-*copia* element Rada reported in the storage protein activator (spa) locus in *Ae. speltoides* and the *T. aestivum* and *T. durum* genomes. Some clones did not reveal any significant similarity to the sequences mentioned for *Ae. speltoides* in the publicly available databases but showed high homology to the RT gene of diploid and allopolyploid wheats, *H. vulgare*, *O. sativa*, *Z. mays*, *B. distachyon*, *S. italica*, *Phyllostachys edulis*, and *P. heterocycla*. Specifically, clones Aesp8, Aesp9, and Aesp10 showed significant homology to the non-Triticeae species, that is, rice, foxtail, false brome, maize, and bamboo, likely representing the evolutionarily ancient TE fraction in the *Ae. speltoides* genome.

Among the Ty3-*gypsy* fragments obtained in this study, the Fatima element was identified in 16 clones, including those containing other TE types (Aesp31/32, Aesp27–Aesp30, and Aesp34) and mitochondrial DNA (Aesp23; Figure 1). All Fatima sequences obtained in this research showed high homology to retrotransposons in Triticeae species. The highly heterogeneous Fatima family composed up to 7% of the TE fraction in the *Ae. speltoides*, which significantly exceeds the abundances of other *gypsy* families [4], and it was highly abundant in the B-genome of allopolyploid wheat [45, 46]. In addition to Fatima, two sequences, Aesp17 and Aesp18, showed significant homology to Carmilla, and the Aesp19 clone was comparable to Nusif retrotransposons in the genome of *T. aestivum*. In contrast to the high abundance in the genome Fatima elements, two similar Ty3-*gypsy-*like fragments, Aesp20 and Aesp21, showed homology only to the Vrn-B1 retrotransposon in the *T. aestivum* genome and the Ty3-*gypsy* retrotransposon in *B. distachyon*. Thus, the *gypsy-*type sequences obtained in this study demonstrated significant nucleotide diversity and different abundances in the *Ae. speltoides* genome and Triticeae species.

Unlike mammalian genomes, non-LTR LINE retrotransposons exhibit low abundance in plants and are much less represented in GenBank than the *copia* and *gypsy* superfamilies. In the *T. aestivum* genome, LINE elements were identified in the subtelomeric DNA marked by the Spelt52 tandem repeat [47], and they comprised 1.3% of chromosome 3B [45]. Three clones, Aesp24 to Aesp26, contained sequences showing homology to the known LINE elements in the genomes of cereals. Clone Aesp24 comprised a Fatima fragment of 72 nt and sequence of 358 nt, which is comparable to the non-LTR retrotransposon in *H. vulgare* (Figure 1). The Aesp25 sequence revealed a homology of 68% to the MIUSE1 retrotransposon in *T. monococcum*. The largest sequence, Aesp26, showed 65–80% similarity to the LINE elements in allopolyploid wheat and *B. distachyon*.

In this study, the CACTA superfamily in the TE fraction of *Ae. speltoides* was represented by clones Aesp22, Aesp31, and Aesp32. These sequences showed identity of 90–97% to the DNA transposon Jorge (CACTA, TIR) in the wheat genome (Supplementary Table S1). Among the total TE content, CACTA elements composed 4.9% of chromosome 3B [45] and were identified in the subtelomeric DNA in *T. aestivum* [47]. In *Ae. speltoides*, Jorge made up 1.84% of the TE content [4].

Most sequences obtained in this research comprised TE fragments of various types (Figure 1). In addition, fragments of Fatima and mitochondrial DNA were identified in clone Aesp23; such a sequence composition points to the TEs' effect on the mitochondrial DNA invasion of the *Ae. speltoides* nuclear genome. The complex Aesp34 clone contained three fragments of *gypsy-*type elements, Fatima, Abilene, and Danae and two fragments of different DNA transposons, *En*/*Spm* and *Harbinger*, flanking a short sequence of 5S rRNA (Figure 1). Part of this sequence, that is, Danae–*En*/*Spm*–5S rRNA–*Harbinger* (540 nt in total), showed an identity of 94% to the genomic scaffold of chromosome 3B of *T. aestivum* and 70% homology to the CKX2.5 gene. The high similarity to chromosome 3B likely evidences nonhomologous recombination and/or mobile element transposition activity event(s) in the diploid B-genome progenitor, resulting in the origin of this complex fragment. The existence of such a comprehensive sequence in the genome is the potential target for illegitimate recombination, in which, specifically, the regular 5S rDNA cluster could be involved. In particular, the insertions of *En*/*Spm* were considered as a potential factor in the 5S rDNA mobility in the genome of *Ae. speltoides* [28, 39]. Furthermore, clone Aesp34 could be evidence for the existence of other evolutionarily conserved complex sequences in the genome and reflect species- and chromosome-specific repetitive DNA patterning/organization.

The fragments of TE sequences obtained in this study, on the one hand, demonstrated dispersed distribution throughout the chromosomes and, on the other, exhibited sequence-specific peculiarities in their patterns. The significant clustering of different TEs was observed in the chromosomal regions adjacent to the 5S rDNA and 45S rDNA loci and in the pericentromeric and distal/terminal positions, that is, chromosome regions corresponding to the Giemsa-positive and tandem repeat-enriched heterochromatic blocks forming species- and chromosome-specific patterns in the *Ae. speltoides* genome [25, 44]. In euchromatin, widely interspersed retrotransposons demonstrate nonrandom and TE-sequence-specific chromosomal clustering [43] (present work, Figure 2), indicating the TEs' impact on the genome architecture and diversification. However, widespread throughout the chromosomes, various TEs and nested mobile elements of different classes and families [11, 46, 48, 49] (present work) are the hotspots for illegitimate recombination (Figures 2(a) and 2(d)), provoking chromosome aberrations in both hetero- and euchromatin regions and eventually leading to genome instability. In contrast, the availability of the same/highly similar sequences in virtually any genome region could prevent the appearance of numerous deleterious DNA lesions, especially in the cases of critical double-strand breaks, as repetitive DNAs—primarily TEs—could serve as the overabundant and ubiquitous templates for nonhomologous DNA repair [50]. Thus, mobile elements perform a dual function in the genome as the main structural fraction of chromatin and, at the same time, a platform for chromosome/genome restructuring under the influence of a variety of internal and external factors, resulting in widespread intraspecific polymorphism in the repetitive DNAs' patterns and abundances in natural populations of *Ae. speltoides* [27, 30].

## 5. Conclusions

In the present research, the genome constitution of wild goatgrass, *Ae. speltoides*, was explored by means of cloning and sequencing different types of mobile elements and cytogenetic analysis of individual TE sequences' chromosomal distributions. The obtained TE clones provided evidence for the enrichments of the genome with different types of TEs, which demonstrated wide nucleotide diversity among sequences of the same superfamily. In addition, widespread intercalating events resulted in complex organization in most of the obtained TE clones. Among the TEs that were found in this study, there were sequences common to Triticeae, as well as sequences showing the similarities of *Ae. speltoides* repetitive DNA fraction to distant genera and reflecting the evolutionary history of the species. Clear differences in the chromosome patterns of individual TE clones will allow these sequences to be used in future studies for understanding the chromosome/genome organization and repatterning under various internal and external impacts.

## Supplementary Materials

Supplementary Materials Supplementary Figure S1: polymerase chain reaction (PCR) amplifications of transposable element (TE) fragments from genomic DNA. Supplementary Table S1: identification of the sequenced transposable element (TE) fragments.
