# Supplementary material for: Transposable Elements in the Organization and Diversification of the Genome of Aegilops speltoides Tausch (Poaceae, Triticeae)
Source: Int J Genomics. 2018 Sep 26;2018:4373089. doi: 10.1155/2018/4373089 (PMC6178165; doi:10.1155/2018/4373089)
Supplement: Supplementary Materials — Supplementary Figure S1: polymerase chain reaction (PCR) amplifications of transposable element (TE) fragments from genomic DNA. Supplementary Table S1: identification of the sequenced transposable element (TE) fragments. [file 4373089.f1.docx]

**Supplementary material**

**Transposable elements in the organization and diversification of the genome of *Aegilops speltoides* Tausch (Poaceae, Triticeae)**

**Olga Raskina**

Institute of Evolution University of Haifa, Aba-Hushi Avenue 199, Mount Carmel, Haifa 498838, Israel

E-mail: [olga@evo.haifa.ac.il](mailto:olga@evo.haifa.ac.il)

**Figure S1:** Polymerase chain reaction (PCR) amplifications of transposable element (TE) fragments from genomic DNA. Individual clones screened for inserts by digestion with *Eco*RI. Genomic mix of PCR-amplified fragments of different lengths of (1) Ty1-*copia,* (4) Ty3-*gypsy*, and (8) LINE retroelements. Individual sequences were cloned, sequenced and PCR-amplified using plasmid DNA as the template and standard T7 and SP6 primers. Individual clones running in 0.9 % agarose gel after *EcoR1* digestion are shown: (2) Aesp2, (3) Aesp7, (5) Aesp16, (6) Aesp18, (7) Aesp21, (9) Aesp29, (10) Aesp, and (11) Aesp26.

**Table S1.** Identification of the Sequenced Transposable Element (TE) Fragments.

| **No.** | **Clone**  **size, nt** | **Sequence identification** | **Highest similarity to:** | **Query cover/**  **Identity, %** |
| --- | --- | --- | --- | --- |
|  | Aesp1  KY404239  284 nt | Ty1-*copia,* WIS ^(1,2,3)^ | [HF541875.1](https://www.ncbi.nlm.nih.gov/nucleotide/478246252?report=genbank&log$=nucltop&blast_rank=2&RID=3ZWEYHEN014) (*Triticum aestivum)*  [AAB48089.1](https://www.ncbi.nlm.nih.gov/protein/1850552?report=genbank&log$=prottop&blast_rank=1&RID=3S8CSFF601R) (*Secale cereale*)  [AAA34262.1](https://www.ncbi.nlm.nih.gov/protein/170678?report=genbank&log$=prottop&blast_rank=3&RID=3S8CSFF601R) (*Triticum aestivum)* | 94 / 94  97 / 83  92 / 84 |
|  | Aesp2  KY404240  284 nt | Ty1-*copia,* WIS ^(1,2,3)^ | [AAB48089.1](https://www.ncbi.nlm.nih.gov/protein/1850552?report=genbank&log$=prottop&blast_rank=1&RID=3S8CSFF601R) (*Secale cereale*)  [AAA34262.1](https://www.ncbi.nlm.nih.gov/protein/170678?report=genbank&log$=prottop&blast_rank=3&RID=3S8CSFF601R) (*Triticum aestivum)* | 97 / 83  92 / 84 |
|  | Aesp3  KY404241  278 nt | Ty1-*copia,* WIS ^(1,2,3)^ | [AAB48089.1](https://www.ncbi.nlm.nih.gov/protein/1850552?report=genbank&log$=prottop&blast_rank=1&RID=3SDCCM8J015) (*Secale cereale*)  [AAA34262.1](https://www.ncbi.nlm.nih.gov/protein/170678?report=genbank&log$=prottop&blast_rank=3&RID=3S8CSFF601R) (*Triticum aestivum)* | 99 / 84  94 / 85 |
|  | Aesp4  KY404242  284 nt | Ty1-*copia,* WIS ^(1,2,3)^ | [D90687.1](http://www.ncbi.nlm.nih.gov/nucleotide/1435007?report=genbank&log$=nucltop&blast_rank=4&RID=W8F15X0F015) ([*Aegilops speltoides*)](http://blast.ncbi.nlm.nih.gov/Blast.cgi#alnHdr_1435007)  [D90632.1](http://www.ncbi.nlm.nih.gov/nucleotide/1434952?report=genbank&log$=nucltop&blast_rank=15&RID=W8F15X0F015) (*Triticum aestivum)* | 85 / 98  85 / 97 |
|  | Aesp5  KY404243  284 nt | Ty1-*copia,* WIS ^(1,2,3)^ | [D90687.1](http://www.ncbi.nlm.nih.gov/nucleotide/1435007?report=genbank&log$=nucltop&blast_rank=4&RID=W8F15X0F015) ([*Aegilops speltoides*)](http://blast.ncbi.nlm.nih.gov/Blast.cgi#alnHdr_1435007)  [D90632.1](http://www.ncbi.nlm.nih.gov/nucleotide/1434952?report=genbank&log$=nucltop&blast_rank=15&RID=W8F15X0F015) (*Triticum aestivum)* | 85 / 98  85 / 97 |
|  | Aesp6  KY404244  280 nt | Ty1-*copia,* WIS ^(1,2,3)^ | [AAB48089.1](https://www.ncbi.nlm.nih.gov/protein/1850552?report=genbank&log$=prottop&blast_rank=1&RID=3S8CSFF601R)(*Secale cereale*)  [AAA34262.1](https://www.ncbi.nlm.nih.gov/protein/170678?report=genbank&log$=prottop&blast_rank=3&RID=3S8CSFF601R)(*Triticum aestivum)* | 88 / 85  88 / 86 |
|  | Aesp7  KY404245  284 nt | Ty1-*copia,* Angela ^(1,2,3)^ | [AAB48089.1](https://www.ncbi.nlm.nih.gov/protein/1850552?report=genbank&log$=prottop&blast_rank=1&RID=3S8CSFF601R) (*Secale cereale*)  [AAA34262.1](https://www.ncbi.nlm.nih.gov/protein/170678?report=genbank&log$=prottop&blast_rank=3&RID=3S8CSFF601R) (*Triticum aestivum)* | 97 / 80  92 / 93 |
|  | Aesp8  KY404246  284 nt | Ty1-*copia,* Rada ^(1,2,3,4)^ | [FM242577.1](https://www.ncbi.nlm.nih.gov/nucleotide/226434270?report=genbank&log$=nucltop&blast_rank=3&RID=3SFPHYUB015) (*Ae. speltoides)*  [DQ890165.1](https://www.ncbi.nlm.nih.gov/nucleotide/117168405?report=genbank&log$=nucltop&blast_rank=2&RID=3SFPHYUB015) (*Triticum aestivum)*  [FM211483.1](https://www.ncbi.nlm.nih.gov/nucleotide/205362430?report=genbank&log$=nucltop&blast_rank=4&RID=8Y4V8NZD016) (*T. durum)*  [T03662](https://www.ncbi.nlm.nih.gov/protein/7444427?report=genbank&log$=prottop&blast_rank=1&RID=3SFPZ9S4014) (Rtr21, *Oryza sativa*)  [LOC_Os02g52020.1](http://rice.plantbiology.msu.edu/cgi-bin/ORF_infopage.cgi?orf=LOC_Os02g52020.1) (*Oryza sativa*)  [AAK84849.1](https://www.ncbi.nlm.nih.gov/protein/15148812?report=genbank&log$=prottop&blast_rank=2&RID=3SFPZ9S4014) (*Zea mays*)  [AF227022.1](https://www.ncbi.nlm.nih.gov/nucleotide/17221851?report=genbank&log$=nucltop&blast_rank=7&RID=8W11Z88701R) (*Setaria adhaerans)* | 95 / 93  95 / 93  93 / 93  97 / 85  96 / 86  97 / 85  93 / 84 |
|  | Aesp9  KY404247  265 nt | Ty1-*copia,* LeojygB ^(1,2,3,4)^ | [HG670306.1](https://www.ncbi.nlm.nih.gov/nucleotide/669026884?report=genbank&log$=nucltop&blast_rank=1&RID=3SGTPD8E01R) (*Triticum aestivum)*  RLC_Hvul_LeojygB_Hn582D21-1(*H.vulgare)* [FO203437.1](https://www.ncbi.nlm.nih.gov/nucleotide/440577297?report=genbank&log$=nucltop&blast_rank=7&RID=3SGTPD8E01R) (*Phyllostachys heterocycla*)  [AAK55320.1](https://www.ncbi.nlm.nih.gov/protein/14164966?report=genbank&log$=prottop&blast_rank=2&RID=3SGU3X4K01R) (*Oryza sativa*)  EMS50149.1 (*Triticum urartu*)  [ADB85257.1](https://www.ncbi.nlm.nih.gov/protein/284434486?report=genbank&log$=prottop&blast_rank=6&RID=3SGU3X4K01R) (*Phyllostachys edulis)*  [LOC_Os01g34490.1](http://rice.plantbiology.msu.edu/cgi-bin/ORF_infopage.cgi?orf=LOC_Os01g34490.1) (*Oryza sativa*) | 97 / 90  100 / 100  97 / 80  99 / 77  97 / 78  97 / 77  97 / 78 |
|  | Aesp10  KY404248  284 nt | Ty1-*copia* ^(1,2,3,4)^ | [HG670306.1](https://www.ncbi.nlm.nih.gov/nucleotide/669026884?report=genbank&log$=nucltop&blast_rank=1&RID=3SHM542W01R) (*Triticum aestivum* chromosome 3B, genomic scaffold)  [AP014963.1](https://www.ncbi.nlm.nih.gov/nucleotide/937924750?report=genbank&log$=nucltop&blast_rank=2&RID=3SHM542W01R) (*Oriza sativa*)  [AAK84842.1](https://www.ncbi.nlm.nih.gov/protein/15148794?report=genbank&log$=prottop&blast_rank=1&RID=3SHMKH0B01R) (*Zea mays)*  [AAL36478.1](https://www.ncbi.nlm.nih.gov/protein/17221888?report=genbank&log$=prottop&blast_rank=4&RID=3SHMKH0B01R) (*Setaria italica*) | 94 / 88  95 / 70  99 / 65  96 / 64 |
|  | Aesp11  KY404249  429 nt | Ty3-*gypsy*, Fatima ^(1,2,3)^ | [FN564432.1](https://www.ncbi.nlm.nih.gov/nucleotide/300681502?report=genbank&log$=nucltop&blast_rank=4&RID=3UXUT60Z014) (*Triticum aestivum*)  [AY204211.1](https://www.ncbi.nlm.nih.gov/nucleotide/28793976?report=genbank&log$=nucltop&blast_rank=2&RID=3UXUT60Z014) ([*Ae. speltoides,* clone Gas-5)](https://blast.ncbi.nlm.nih.gov/Blast.cgi#alnHdr_28793976)  [AY103468.1](https://www.ncbi.nlm.nih.gov/nucleotide/21464516?report=genbank&log$=nucltop&blast_rank=3&RID=3UXUT60Z014) (Gas-3)  [AY204210.1](https://www.ncbi.nlm.nih.gov/nucleotide/28793975?report=genbank&log$=nucltop&blast_rank=5&RID=3UXUT60Z014) (Gas-4)  [AY204209.1](https://www.ncbi.nlm.nih.gov/nucleotide/28793974?report=genbank&log$=nucltop&blast_rank=6&RID=3UXUT60Z014) (Gas-2)  [AY204208.1](https://www.ncbi.nlm.nih.gov/nucleotide/28793973?report=genbank&log$=nucltop&blast_rank=7&RID=3UXUT60Z014) (Gas-1) | 93 / 95  93 / 95  93 / 95  93 / 94  93 / 94  92 / 93 |
|  | Aesp12  KY404250  503 nt | Ty3-*gypsy*, Fatima ^(1,2,3)^ | [FN564432.1](https://www.ncbi.nlm.nih.gov/nucleotide/300681502?report=genbank&log$=nuclalign&blast_rank=2&RID=3V5HAVYJ014) (*Triticum aestivum* ) | 97 / 93 |
|  | Aesp13  KY404251  429 nt | Ty3-*gypsy*, Fatima ^(1,2,3)^ | [FN564432.1](http://www.ncbi.nlm.nih.gov/nucleotide/300681502?report=genbank&log$=nucltop&blast_rank=5&RID=VUTFUP3501R) (*Triticum aestivum*)  Clones Gas-1 to Gas-5 | 89 / 95  90-93/ 92-97 |
|  | Aesp14  KY404252  361 nt | Ty3-*gypsy*, Fatima ^(1,2,3)^ | [AH012974.2](http://www.ncbi.nlm.nih.gov/nucleotide/1015633048?report=genbank&log$=nuclalign&blast_rank=16&RID=WUPNB8CR015) (*Aegilops tauschii* ) | 98 / 82 |
|  | Aesp15  KY404253  1006 nt | Ty3-*gypsy*, Fatima ^(1,2,3)^ | [HF541876.1](https://www.ncbi.nlm.nih.gov/nucleotide/478246253?report=genbank&log$=nucltop&blast_rank=4&RID=09C13TJ3014)(*Triticum aestivum*) | 99 / 98 |
|  | Aesp16  KY404254  381 nt | Ty3-*gypsy*, Fatima ^(1,2,3)^ | [EF426565.1](https://www.ncbi.nlm.nih.gov/nucleotide/133741922?report=genbank&log$=nucltop&blast_rank=2&RID=09F6RRDY016) *(Triticum aestivum )* | 91/ 90 |
|  | Aesp17  KY404255  505 nt | Ty3-*gypsy*, Carmilla ^(1,2,3)^ | [HF541871.1](https://www.ncbi.nlm.nih.gov/nucleotide/478246247?report=genbank&log$=nucltop&blast_rank=3&RID=093VSMXS01R) *(Triticum aestivum)* | 93 / 78 |
|  | Aesp18  KY404256  502 nt | Ty3-*gypsy*, Carmilla ^(1,2,3)^ | [FN564431.1](https://www.ncbi.nlm.nih.gov/nucleotide/300681497?report=genbank&log$=nuclalign&blast_rank=2&RID=07PBXZXK014) *(Triticum aestivum)* | 92 / 81 |
|  | Aesp19  KY404257  223 nt | Ty3-*gypsy*, Nusif ^(1,2,3)^ | [FN564430.1](https://www.ncbi.nlm.nih.gov/nucleotide/300681466?report=genbank&log$=nuclalign&blast_rank=4&RID=3V1CZ9WY015) *(Triticum aestivum)* | 88 / 79 |
|  | Aesp20  KY404258  997 nt | Ty3-*Gypsy,* Vrn-B1 ^(1,3)^ | [HQ186251.1](https://www.ncbi.nlm.nih.gov/nucleotide/323134588?report=genbank&log$=nucltop&blast_rank=3&RID=047ZWNTU014) (*Triticum aestivum*) [JN817430.1](https://www.ncbi.nlm.nih.gov/nucleotide/375333766?report=genbank&log$=nuclalign&blast_rank=2&RID=YM81RWHT014) (*Triticum carthlicum*)  ADDN01001033; Gypsy-37_BD-I repbase  (*Brachypodium distachyon*) | 89 / 68  89 / 68  91 / 61 |
|  | Aesp21  KY404259  1001 nt | Ty3-*Gypsy,* Vrn-B1 ^(1,3)^ | [HQ186251.1](https://www.ncbi.nlm.nih.gov/nucleotide/323134588?report=genbank&log$=nucltop&blast_rank=3&RID=047ZWNTU014) (*Triticum aestivum*)[JN817430.1](https://www.ncbi.nlm.nih.gov/nucleotide/375333766?report=genbank&log$=nuclalign&blast_rank=2&RID=YM81RWHT014) (*Triticum carthlicum*) ADDN01001033; Gypsy-37_BD-I repbase (*Brachypodium distachyon*) | 89 / 68  89 / 68  88 / 66 |
|  | Aesp22  KY404260  202 nt | CACTA (TIR), Jorge ^(1,2)^ | [FN564427.1](https://www.ncbi.nlm.nih.gov/nucleotide/300681419?report=genbank&log$=nuclalign&blast_rank=2&RID=07JXF19T016) (Triticum aestivum) | 79 / 97 |
|  | Aesp23  KY404261  557 nt | Ty3-*Gypsy;* mitochondrial DNA ^(1,3)^ | [AP013107.1](https://www.ncbi.nlm.nih.gov/nucleotide/549067759?report=genbank&log$=nucltop&blast_rank=1&RID=06DD6ARA014) *(Ae. speltoides* mitochondrial DNA)[AP013106.1](https://www.ncbi.nlm.nih.gov/nucleotide/549067723?report=genbank&log$=nucltop&blast_rank=2&RID=096Z4W48014) (*T.timopheevi* mitochondrial DNA)[EU534409.1](https://www.ncbi.nlm.nih.gov/nucleotide/169649045?report=genbank&log$=nucltop&blast_rank=3&RID=096Z4W48014) *(T. aestivum* mitochondrial DNA) ANPC01003415; Gypsy-5_Cia-I repbase; Gypsy-8_Cia-I repbase (*Cicer arietinum*) | 94/ 98  94 / 98  94 / 98  Similarity 0.7-0.8 |
|  | Aesp24  KY404262  460 nt | Non-LTR, LINE ^(1,3)^ | [CR626934.1](https://www.ncbi.nlm.nih.gov/nucleotide/61656803?report=genbank&log$=nucltop&blast_rank=4&RID=408R04DR015) ([*Triticum aestivum*](https://blast.ncbi.nlm.nih.gov/Blast.cgi#alnHdr_109450933))  [CT009735.1](https://www.ncbi.nlm.nih.gov/nucleotide/109450933?report=genbank&log$=nucltop&blast_rank=3&RID=408R04DR015) ([*Triticum aestivum*](https://blast.ncbi.nlm.nih.gov/Blast.cgi#alnHdr_109450933))  [CT009625.1](https://www.ncbi.nlm.nih.gov/nucleotide/109450922?report=genbank&log$=nuclalign&blast_rank=6&RID=408R04DR015) (*Aegilops tauschii*)  [AAG13524.1](https://www.ncbi.nlm.nih.gov/protein/10140689?report=genbank&log$=protalign&blast_rank=3&RID=408R9GUZ015) (*Oryza sativa*) reverse transcriptase | 88 / 66  88 / 66  95 / 65  83 / 42 |
|  | Aesp25  KY404263  384 nt | Non-LTR, LINE, MIUSE1^(1,3)^ | [FN564426.1](https://www.ncbi.nlm.nih.gov/nucleotide/299109310?report=genbank&log$=nuclalign&blast_rank=2&RID=0F19AXUX01R) (*Triticum aestivum*)  [AF459639.1](https://www.ncbi.nlm.nih.gov/nucleotide/18496650?report=genbank&log$=nuclalign&blast_rank=3&RID=40EVT6J9015) (*Triticum monococcum*) | 98 / 67  81 / 68 |
|  | Aesp26  KY404264  710 nt | Non-LTR, LINE ^(1,3)^ | [L1-12_BDi](http://www.girinst.org/protected/repbase_extract.php?access=L1-12_BDi&format=EMBL) (*Brachypodium distachyon*)  [L1-6_BDi](http://www.girinst.org/protected/repbase_extract.php?access=L1-6_BDi&format=EMBL) (*Brachypodium distachyon*)  [FN564430.1](https://www.ncbi.nlm.nih.gov/nucleotide/300681466?report=genbank&log$=nuclalign&blast_rank=2&RID=0F46AUYC014) (*Triticum aestivum*) | Similarity 0.65  Similarity 0.80  97 / 69 |
|  | Aesp27  KY404265  512 nt | Ty1-*copia,* Barbara; Ty3-*gypsy,* Fatima ^(1,2,3)^ | [JX978695.1](https://www.ncbi.nlm.nih.gov/nucleotide/411113262?report=genbank&log$=nuclalign&blast_rank=2&RID=465XMFKP014) (*Triticum urartu*)  [FM242577.1](https://www.ncbi.nlm.nih.gov/nucleotide/226434270?report=genbank&log$=nuclalign&blast_rank=31&RID=0C3FDAV801R) (*Ae.speltoides*)  HE774676.1 (*Triticum aestivum*)  [FN564431.1](https://www.ncbi.nlm.nih.gov/nucleotide/300681497?report=genbank&log$=nuclalign&blast_rank=16&RID=487TUUXB014) (*Triticum aestivum*) | 94 / 94  77 / 89  90 / 96  97 / 85 |
|  | Aesp28  KY404266  509 nt | Ty1-*copia,* Barbara; Ty3-*gypsy,* Fatima ^(1,2,3)^ | FN564429.1 (*Triticum aestivum*) [JX978695.1](https://www.ncbi.nlm.nih.gov/nucleotide/411113262?report=genbank&log$=nuclalign&blast_rank=2&RID=465XMFKP014) (*Triticum urartu* )  [FN564431.1](https://www.ncbi.nlm.nih.gov/nucleotide/300681497?report=genbank&log$=nuclalign&blast_rank=16&RID=487TUUXB014) (*Triticum aestivum*) | 95 / 94  95 / 94  97 / 85 |
|  | Aesp29  KY404267  512 nt | Ty1-*copia,* Barbara; Ty3-*gypsy,* Fatima ^(1,2,3)^ | [FN564431.1](https://www.ncbi.nlm.nih.gov/nucleotide/300681497?report=genbank&log$=nuclalign&blast_rank=16&RID=487TUUXB014) (*Triticum aestivum*) | 95 / 86 |
|  | Aesp30  KY404268  512 nt | Ty1-*copia,* Barbara; Ty3-*gypsy,* Fatima ^(1,2,3)^ | [FN564431.1](https://www.ncbi.nlm.nih.gov/nucleotide/300681497?report=genbank&log$=nuclalign&blast_rank=16&RID=487TUUXB014) (*Triticum aestivum*) | 97 / 85 |
|  | Aesp31  KY404269  617 nt | Ty3-*gypsy,* Fatima;  CACTA (TIR), Jorge ^(1)^ | [AM932686.1](https://www.ncbi.nlm.nih.gov/nucleotide/194239077?report=genbank&log$=nuclalign&blast_rank=26&RID=47XN5WYB015) (*Triticum aestivum*)  [FN564432.1](https://www.ncbi.nlm.nih.gov/nucleotide/300681502?report=genbank&log$=nucltop&blast_rank=2&RID=47WRE9BV014) (*Triticum aestivum*) | 96 / 93  91/ 90 |
|  | Aesp32  KY404270  605 nt | Ty3-*gypsy*, Fatima^(1,2,3)^  CACTA (TIR), Jorge ^(1)^ | [AM932686.1](https://www.ncbi.nlm.nih.gov/nucleotide/194239077?report=genbank&log$=nuclalign&blast_rank=26&RID=47XN5WYB015) (*Triticum aestivum*)  [FN564432.1](https://www.ncbi.nlm.nih.gov/nucleotide/300681502?report=genbank&log$=nucltop&blast_rank=2&RID=47WRE9BV014) (*Triticum aestivum*) | 90 / 90  98 / 93 |
|  | Aesp33  KY404271  491 nt | Ty3-*gypsy*, Fatima ^(1,2,3)^ | [FN645450.1](https://www.ncbi.nlm.nih.gov/nucleotide/300681572?report=genbank&log$=nucltop&blast_rank=2&RID=48AR2W0301R) (*Triticum aestivum*) [HF541872.1](https://www.ncbi.nlm.nih.gov/nucleotide/478246248?report=genbank&log$=nucltop&blast_rank=3&RID=48AR2W0301R) (Triticum aestivum) | 96 / 89  96/ 87 |
|  | Aesp34  KY404272  942 nt | Ty3-*gypsy*, Fatima, Abilene, Danae;  CACTA, En/Spm;  5S rRNA;  Harbinger (TIR) ^(1,3)^ | [HG670306.1](https://www.ncbi.nlm.nih.gov/nucleotide/669026884?report=genbank&log$=nuclalign&blast_rank=1&RID=2K49HK0S014) (*Triticum aestivum*)  AF326781;[FATIMA1_TM_LTR](http://www.girinst.org/protected/repbase_extract.php?access=FATIMA1_TM_LTR&format=EMBL)  AC216571;[Gypsy-8_TA-LTR](http://www.girinst.org/protected/repbase_extract.php?access=Gypsy-8_TA-LTR&format=EMBL)  En/Spm, CACTA; [EnSpm-17_SBi](http://www.girinst.org/protected/repbase_extract.php?access=EnSpm-17_SBi&format=EMBL)  [HG670306.1](https://www.ncbi.nlm.nih.gov/nucleotide/669026884?report=genbank&log$=nuclalign&blast_rank=1&RID=2K49HK0S014) 5S rRNA,  (from 432059562 to 432059755)  Harbinger, TIR; [Harbinger-3_LCh](http://www.girinst.org/protected/repbase_extract.php?access=Harbinger-3_LCh&format=EMBL) | 100 / 84 %  Similarity 0.93  Similarity 0.74  Similarity 0.82  100 / 81  Similarity 0.76 |

1 – NCBI (<https://blast.ncbi.nlm.nih.gov/Blast.cgi>)

2 – TREP (<http://botserv2.uzh.ch/kelldata/trep-db/index.html>)

3 – GIRI (<http://www.girinst.org/>)

4 – TIGR (<http://rice.plantbiology.msu.edu/index.shtml>)
